# Sub-Millisecond Response Time in a Photorefractive Composite Operating under CW Conditions

**DOI:** 10.1038/srep30810

**Published:** 2016-08-01

**Authors:** Jong-Sik Moon, Tyler E. Stevens, Todd C. Monson, Dale L. Huber, Sung-Ho Jin, Jin-Woo Oh, Jeffrey G. Winiarz

**Affiliations:** 1 Department of Chemistry, Missouri University of Science and Technology, Rolla, MO 65409, USA; 2 BK21 plus Division of Nano Convergence Technology, Pusan National University, Busan 46241, Korea; 3 Sandia National Laboratories, P.O. Box 5800, Albuquerque, NM 87112, USA; 4 Department of Chemistry Education, Graduate Department of Chemical Materials, and Institute for Plastic Information and Energy Materials, Pusan National University, Busan 46241, Korea; 5 Department of Nano energy Engineering, Pusan National University, Busan 46241, Korea

## Abstract

Extensive study of photorefractive polymeric composites photosensitized with semiconductor nanocrystals has yielded data indicating that the inclusion of such nanocrystals enhances the charge-carrier mobility, and subsequently leads to a reduction in the photorefractive response time. Unfortunately, the included nanocrystals may also act as a source of deep traps, resulting in diminished diffraction efficiencies as well as reduced two beam coupling gain coefficients. Nonetheless, previous studies indicate that this problem is mitigated through the inclusion of semiconductor nanocrystals possessing a relatively narrow band-gap. Here, we fully exploit this property by doping PbS nanocrystals into a newly formulated photorefractive composite based on molecular triphenyldiamine photosensitized with C_60_. Through this approach, response times of 399 *μ*s are observed, opening the door for video and other high-speed applications. It is further demonstrated that this improvement in response time occurs with little sacrifice in photorefractive efficiency, with internal diffraction efficiencies of 72% and two-beam-coupling gain coefficients of 500 cm^−1^ being measured. A thorough analysis of the experimental data is presented, supporting the hypothesized mechanism of enhanced charge mobility without the accompaniment of superfluous traps. It is anticipated that this approach can play a significant role in the eventual commercialization of this class of materials.

Owing to their large optical nonlinearities, low permittivity and low cost, organic photorefractive (PR) composites are potentially useful in a variety of real-time optical applications^1^,^2^,^3^,^4^,^5^. Polymer-based PR composites are attractive due to the ease with which their constituents may be independently modified, allowing the composite to be tailored for a specific application. Despite the flexibility associated with organic PR composites, the lack of organic photosensitizers exhibiting sufficiently large photo-charge generation at near infrared wavelengths, *λ*, proved to be a significant hurdle. It was primarily this challenge which inspired the photosensitization of otherwise all-organic PR composites through the inclusion of semiconductor nanocrystals, also known as *quantum dots* or *Q-dots*^6^,^7^,^8^,^9^,^10^,^11^,^12^,^13^,^14^,^15^,^16^,^17^,^18^,^19^,^20^,^21^,^22^,^23^. Although motivated by the ability to extend the spectral range accessible to this class of materials, most studies have been conducted at visible wavelengths, primarily *λ* = 633 nm, using Q-dots such as nano-sized CdS (QCdS) as well as QCdSe and QCdTe^6^,^7^,^9^,^10^,^11^,^12^,^13^,^14^,^17^,^20^,^21^,^22^. Although far fewer examples exist, narrower band-gap semiconductor materials such as QPbS or QPbSe have been generally used for *λ* such as 1.31 *μ*m and 1.51 *μ*m, however, reported efficiencies and response times have been substandard relative to those reported for PR composites photosensitized with more traditional organics, most notably C_60_^8^,^15^,^16^,^23^.

In addition to acting as a photosensitizer, a significant amount of experimental data, particularly from time-of-flight characterizations, indicate the included Q-dots also increase the charge-carrier mobility, *μ*, within the PR composite^10^. This enhancement in *μ* is primarily attributed to the ability of the free charge-carriers to enter into, and subsequently be transported through the included Q-dots, where they experience a faster *μ* relative to that attributed to the organic charge-transporting species. Not surprisingly, it has also been demonstrated that this improvement in the *μ* may be accompanied by a reduction in the PR response time, *τ*^17^,^18^,^23^. Notwithstanding the enormous insights gained into the fundamental processes of charge-generation and charge-transport occurring in inorganic-organic hybrid composites, there has existed an inability to capitalize on these understandings with, until recently, *τ* > 100 ms representing the best PR response time reported for an organic composite photosensitized with Q-dots^17^,^23^. This *τ* is significantly larger than those reported for PR composites photosensitized with organic photosensitizers such as C_60_^24^,^25^,^26^,^27^,^28^,^29^,^30^,^31^,^32^,^33^,^34^,^35^. Furthermore, for studies involving visible *λ*, the data suggest that while the inclusion of Q-dots introduces a secondary charge-transport species imparting enhanced *μ*, they may also act as a trapping species^17^,^18^,^23^. While a certain concentration and depth of traps is necessary for the PR effect, excessive trapping of positive free charge-carriers can have a detrimental influence over the *τ*, operational voltage, *E*, as well as the overall diffraction efficiency^1^,^2^,^3^,^36^. In an effort to circumvent this issue, a recent study investigated the photosensitization of a PR composite at *λ* = 633 nm through the inclusion of QPbS where the QPbS exhibited significant, but off-resonance, absorption^23^. In a separate study it was established that by exchanging the relatively wide band-gap QCdSe with QCdTe, which possesses a comparatively narrower band-gap, an improvement in the *τ* was realized^17^. These studies provided strong evidence that in addition to the photosensitization of PR composites, Q-dots may alternatively be used specifically for enhancing the *μ* and thereby substantially reducing the *τ* of a PR composite.

In this communication we present a novel PR composite specifically designed to exploit the insights gained with regard to the influence of Q-dot inclusion over charge-carrier *μ* and subsequent reduction of the *τ*. In many cases, organic PR composites described in literature use poly(*N*-vinylcarbazole) (PVK) due to its efficient hole-transporting capability. More recently PR composites based on polymers which replace the charge-transporting carbazole group with that of triphenyldiamine (TPD), or similar structures, have been described^26^,^27^,^28^,^29^,^30^,^31^,^32^,^33^,^35^. Of these polymers perhaps the most notable is poly(acrylic tetraphenyldiaminobiphenyl) (PATPD) which is composed of a TPD molecule attached to acrylic backbone through a pendant linkage^28^,^30^,^31^,^32^,^33^. In this study, a molecular form of TPD acts as the primary organic charge-transport species. Through this approach, the inert backbone, as well as the pendant linkage associated with polymerized forms of TPD can be eliminated. Furthermore, there is no need for the addition of an inert plastiziser, greatly increasing the loading content of the active components and thereby improving the PR performance. Finally, it is noted that by using a molecular charge-transport species, the conformational traps associated with a polymeric charge-transport species are absent. Conformational traps are known to be relatively shallow in nature and therefore do not contribute significantly to the establishment of a space-charge field^37^. It has been shown, however, that the ellimination of shallow traps can result in an enhancement in the PR *τ*^38^.

In addition to the charge-transporting molecular TPD, the composite described herein also contains the non-linear optical (NLO) dye 4-homopiperidinobenzylidenemalononitrile (7-DCST) providing the electro-optic activity required for the PR effect^25^. 7-DCST has been used in conjunction with PVK as well as PATPD and is a common choice of NLO dye for PR composites in which the optimization of *τ* is of interest^24^,^25^,^30^,^31^,^32^,^33^. Solid phase crystallization of the molecular TPD as well as that of the 7-DCST, also well known for its susceptibility to phase-separation, was successfully suppressed through the inclusion of a small amount of polymer. Rather than employ a functionally passive polymer, it was found that PVK in concentrations as low as 10 wt% was especially effective at preventing detectable aggregation of the molecular constituents. Since the primary goal of this study was to study the performance of a PR composite in which the charge-carrier *μ* is enhanced through the inclusion of narrow band-gap Q-dots, there was no need for the Q-dots to perform as the photosensitizer as in most of the previous studies involving the inclusion of Q-dots in an otherwise all-organic PR composite. This, in conjunction with the experimental *λ* = 633 nm, permitted the use of C_60 _as the photosensitizer in the current study. To study the enhancement of the *τ* as a result of the inclusion of narrow band-gap Q-dots, QPbS was introduced into the previously described composite at various concentrations. It will be demonstrated that the inclusion of the QPbS in the PR composite resulted in a decrease in *τ* by a factor of 6, to *τ* = 399 *μ*s, the fastest *τ* reported for PR composite under CW conditions. This significant decrease in *τ* is especially exciting due to the ability to avoid the detrimental effect over PR efficiency, with *η*_ext_ decreasing by only 4%, to 59%, as a result of introducing the QPbS into the composite.

To characterize the performance of the composites, time-resolved degerate-four-wave-mixing (DFWM), two-beam-coupling (TBC), visible absorption spectroscopy, and conductivity experiments were employed, the results and implications of which will be presented. In addition to the insights gained into the fundamental mechanisms relevant to this class of materials, a significant advancement in PR performance is realized. It is anticipated that the advances illustrated herein represent significant progress towards the eventual practical application of PR composite materials.

## Results

The goal of this study is to examine the feasibility of using narrow band-gap Q-dots to affect charge-carrier *μ* in a PR composite so as to improve the *τ* without detrimentally affecting the PR efficiency of the composite. The first objective to these ends involved the choice of semiconductor material^17^,^18^,^23^. In this regard, it is noted that in all previous studies concerning the inclusion of Q-dots in a PR composite, the Q-dots were included to serve as the photosensitizer. However, data accumulated in these studies demonstrate that in addition to creating free charge-carriers within the photoconductive (PC) matrix, the Q-dots also enhance the *τ* of the PR composites. It was speculated that this enhancement in *τ* was attributable to the faster charge-carrier *μ* associated with inorganic semiconductors as compared to the organic matrix, typically PVK. Definitive evidence of the charge-carrier *μ* being enhanced in a PC polymer through the inclusion of Q-dots came in a study in which QCdS was doped into a thin film of PVK^11^. Using time-of-flight experimental techniques, the QCdS/PVK composite exhibited a faster charge carrier *μ* than undoped PVK. Moreover, it was demonstrated that the *μ* scaled with Q-dot concentration.

Despite these promising results, there is also evidence that Q-dots may play a third role in addition to photocharge-generator and charge-transport species, that of a charge trapping species. Although a certain concentration and depth of charge traps are required for the PR effect, impurities and imperfections associated with the polymer have traditionally fulfilled this requirement, and a separate component is not usually included. For the case of Q-dots, data indicate that their propensity to act as charge traps can have a detrimental influence over the efficiency of a PR composite into which they are included. In an effort to overcome this hindrance, recent work has focused on the use of Q-dots with a narrower band-gap^24^. These studies have demonstrated the ability to photosensitize a PR polymeric composite with QPbS where, unlike in the majority previous studies, the QPbS had a band-gap energy (1.02 eV) which was considerably less than that associated with the optical radiation employed in the PR characterizations (*λ* = 633 nm, corresponding to 1.96 eV). Here, using the relatively narrow band-gap QPbS resulted in *τ* = 34 ms, which is among the fastest reported for a polymeric PR composite photosensitized with Q-dots, as well as for polymeric PR composites in general. In addition, this *τ* was achieved without sacrificing efficiency, as indicated by the internal diffraction effciency, *η*_int_ = 83% reported for this PR composite. Based on these results, it was determined that QPbS would serve as the Q-dot used in the current study. It is noted that the QPbS used in the current study originate from the same batch as those used in this cited work and were passivated with oleic acid (OA) and 1-octadecene (ODE)^23^.

TEM images of the QPbS were obtained and can be found elsewhere^23^. The particles are irregularly shaped, but approximately spherical with a the mean diameter of 3.62 ± 0.58 nm. The UV-Vis absorption spectrum of the QPbS dispersed in toluene was also collected, showing that the first exciton occurs at ~1220 nm^23^. At a normalized concentration of 1 mg/mL, the QPbS exhibits an absorption coefficient of *α*_1220_ = 0.0296 cm^−1^ at 1220 nm and *α*_633_ = 0.234 cm^−1^ at 633 nm. The presence of the well defined peak at ~1220 nm also indicates a high degree of monodispersity. To quantify the ratio of organic capping material to inorganic PbS present in the QPbS, TGA was conducted^24^. In summary, the inorganic PbS accounts for 33.6 wt% of the QPbS, with the remainder of the mass being primarilly attributed to OA and ODE.

The next objective in this study was the formulation of the organic components constituting the PR composite. Because this study is largely concerned with the PR *τ*, it was this parameter which motivated the choices made in this regard. Perhaps the most influential component over the PR *τ* is that of the charge-transporting species. This functionality is often provided through the inclusion of a PC polymer, with PVK being one of the most prevalent. More recent research indicates, however, that PR composites which contain TPD as the charge-transporting moiety exhibit superior PR performance compared to their PVK counterparts, attributed in part to a faster charge-carrier *μ* associated with TPD^26^,^27^,^28^,^29^,^30^,^31^,^32^,^33^. The superior performance associated with TPD-based composites can also be traced to the relationship among the various highest-occupied-molecular-orbitals (HOMO) associated with the components, especially when used in conjunction with certain NLO dyes^28^. Specifically, TPD is more easily ionized than is PVK since the HOMO of TPD lies higher in energy at approximately −5.4 eV, than that of PVK which occurs at −5.92 eV. Furthermore, when PVK is used in conjunction with certain NLO dyes, such as 7-DCST which has a HOMO occurring at −5.9 eV, it is energetically favorable for the positively charged holes to become trapped at a 7-DCST molecule, whereas in the case of TPD this issue is avoided^28^. It is also noted that because the HOMO of TPD is higher in energy than that of the HOMO in PVK, it is energetically favorable for photogenerated holes to remain associated with the TPD and therefore it is not anticipated that the PVK plays any significant role in the charge-transport process.

The TPD-based PR composites typically contain polymers in which the TPD is either part of the polymer backbone or alternatively as a pendant unit grafted to a polymer backbone. When employed in the pendant geometry as in PATPD, TPD-based PR composites have exhibited *τ* = 10 ms with *η*_int_ = 80% under CW conditions. These figures-of-merit are remarkable, especially considering that PATPD is 78.9 wt % TPD, with the remainder of the mass being attributable to the polymer backbone and pendant structure, which is essentially inert with respect to the PR process. Furthermore, PATPD has a *T*_g_ well above RT, dictating the need to include an inert plasticizer allowing for the orientational mobility of the NLO dye. There are also questions as to whether the TPD pendants are able to achieve the optimal stacking conformation which is conducive to the hopping mechanism of charge-transport ubiquitous to these types of PC materials. Faster *τ* have been reported for other PR composites utilizing polymerized forms of TPD, however these fast *τ* often come at the expense of efficiency. Several studies exist in which *τ* as fast as 1–3 ms are reported, though diffraction efficiencies were either not reported or did not exceed 0.5%^26^,^29^. To date, very few examples of a sub-millisecond *τ* have been reported for PR composites characterized under CW conditions^35^. A sub-millisecond of *τ* = 300 *μ*s has been described for a PR composite characterized under pulse conditions^33^.

Based on these considerations, a PR composite rooted in a molecular form of TPD is desirable. Nevertheless, polymeric forms of TPD are typically employed due to the very large propensity for TPD, as well as its various molecular derivatives, to crystallize within the solid solution of the PR composite. After screening a substantial number of TPD derivatives in conjunction with several NLO dyes as well as selected organic photosensitizers, it was established that a PR composite, stable against aggregation of any components for at least 12 months up until now, could be achieved using the N,N-Bis(3-methylphenyl)-N,N-bis(phenyl)benzidine (DMTPD) derivative of TPD as the PC species. Although fraught with a pair of methyl groups to aid in phase stability, this is a relatively small cost in terms of additional inert mass. As will be discussed in greater detail, this allowed for ~45 wt% loading of DMTPD, a relatively high concentration that is favorable for charge-carrier *μ*. In addition to the DMTPD, a relatively small amount of PVK totaling ~10 wt% was introduced to assist in phase stabilization. Although difficult to confirm, it is likely that charge-transport occurs in the PVK as well as the DMTPD, and as such the PVK may not be inert with regard to the PR process. However, based on the HOMO considerations, a positively charged hole is lower in energy when associated with DMTPD than when with PVK, and as such, once a hole becomes associated with DMTPD, it is unlikely to re-associate with the PVK.

To impart NLO activity to the PR composite, 7-DCST was employed as it has been in many recent studies which have highlighted the PR *τ*^24^,^25^,^30^,^31^,^32^,^33^,^34^,^35^. The PR composites described herein contained ~45 wt% 7-DCST which is similar to concentrations employed in previously described PR composites. The photosensitizer used in the current study was C_60_ and is well known to be one of the most efficient PR photosensitizers for *λ* = 633 nm. With a HOMO occurring at −6.40 eV, C_60_ can also behave as a trapping species within the PR medium. In previous studies C_60_ has been used in a range of concentrations, but typically on the order of 0.2–2.0 wt%. In this study C_60_ was included at 0.2 wt% which is a relatively low concentration. Although attempts were made to increase the C_60_ concentration in an effort to improve the PR performance, it was observed that unlike DMTPD and 7-DCST, C_60_ exhibited a very high susceptibility to aggregation within the PR composite at higher concentrations. The resulting PR device is referred to as DC60PBS and its composition is detailed in Table 1.

**Figure 1:**
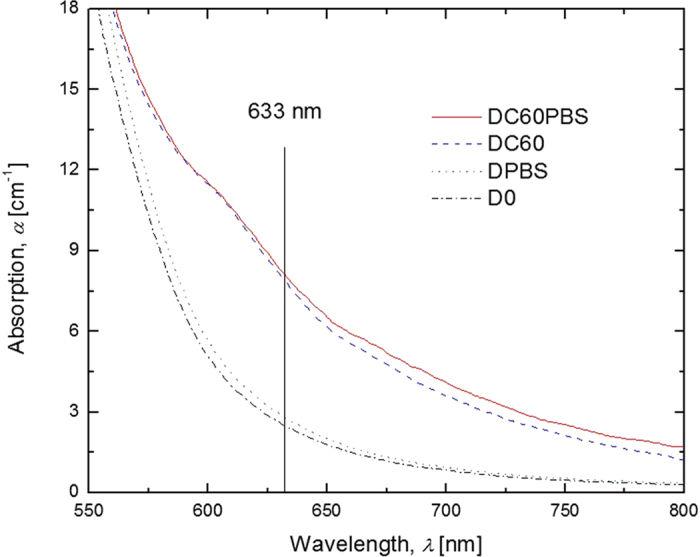
Visible absorption spectra of the PR devices used in this investigation; DC60PBS (solid line), DC60 (dashed line), and DPBS (dotted line).

As described in the Experimental section several control devices were also fabricated and their compositions as well as the *α*_633_ are also detailed in Table 1. The absorption spectra of the devices employed in this study are provided in Fig. 1. The device void of C_60_ and QPbS, D0, exhibits *α*_633_ = 2.46 cm^−1^ whereas the addition of 0.223 wt % of QPbS, as in the DPBS device, increased the *α*_633_ by 0.29 cm^−1^. Similarly, the addition of 0.200 wt % of C_60_, as in DC60, resulted in an increase in *α*_633_ of 5.35 cm^−1^. Finally, in the DC60PBS device, the presence of both C_60_ and QPbS at concentrations commensurate with those of the control devices produced an increase in *α*_633_ by 5.58 cm^−1^.

**Figure 2:**
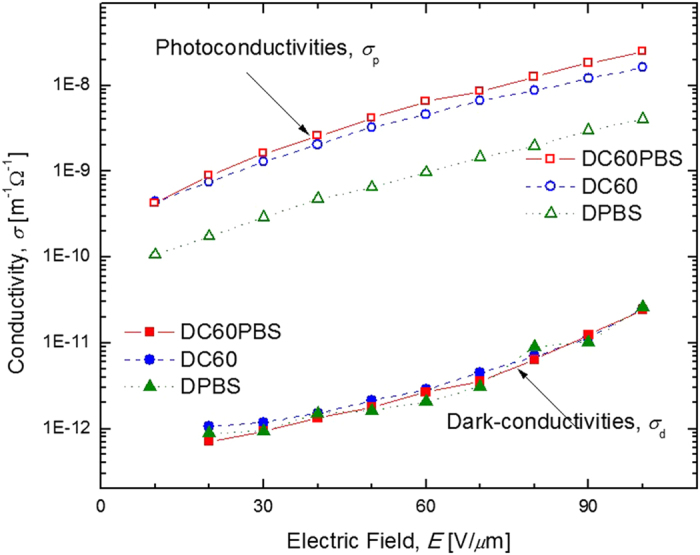
Electric field dependence of the photoconductivities, *σ*_p_, (open symbols) and dark-conductivities, *σ*_d_, (filled symbols) of DC60PBS (squares), DC60 (circles), and DPBS (triangles) at *λ* = 633 nm. The lines are guides for the eye.

For the DC60PBS device, C_60_ is intended as the primary sensitizer, however, QPbS, included for purposes of enhanced charge-transport, has also been shown to photosensitize similar composites with *λ* = 633 nm^24^. In an effort to distinguish between the effects of the respective potential photosensitizers as well as to gain insight in the fundamental physical processes occurring in these materials, the photoconductivity, *σ*_p_, and dark conductivity, *σ*_d_, were measured for each device and are shown as a function of *E* in Fig. 2. Looking first at the *σ*_d_, it is observed that this parameter is nearly constant for all devices across the entire range of investigated *E*. While somewhat surprising, it indicates that the presence of C_60_ and/or QPbS does not have a significant effect on the *σ*_d_. Because conductivities in general, *σ*, depend explicitly on mobility as


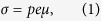


where *p* is the density of mobile charge carriers (holes) and *e* is the fundamental electric charge, it can be assumed that the unilluminated QPbS does not enhance charge carrier mobility under these conditions. This situation is favorable for the PR effect because the establishment of a space-charge field depends on large ratio of *σ*_p_/*σ*_d_. Turning to the *σ*_p_, it is apparent from the figure that the DC60PBS device exhibits the best performance in this regard. This is not surprising since this composite contains both C_60_ and QPbS. The DC60 device shows a slightly diminished *σ*_p_ relative to that of the DC60PBS device. This is again expected since DC60, though void of QPbS, still contains the primary photosensitizer, C_60_. The DPBS device, although decreased by nearly an order of magnitude relative to the DC60PBS device, still exhibits a significant increase *σ*_p_ relative to its *σ*_d_, again demonstrating the ability for QPbS for photosensitize this composite, though significantly less effectively than that of the C_60_ at the relevant concentrations. It is noted that for this study the concentration of QPbS was chosen so as to optimize the *τ* of the DC60PBS device and not the *σ*_p_ of the DPBS device, and therefore it is not possible to draw a direct comparison of the photosensitization ability between the QPbS and C_60_ from these data. The DC0 device (not shown in the figure) did not exhibit a measurable *σ*_p_.

**Figure 3:**
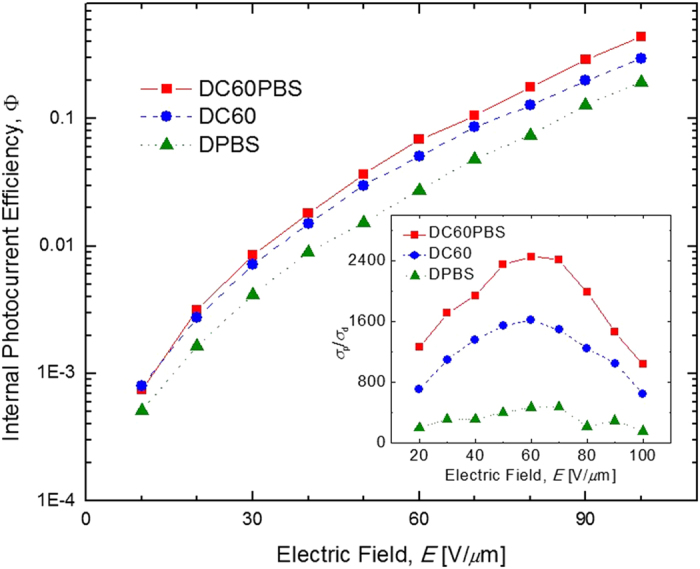
Electric field dependence of the internal photocurrent efficiencies, Φ, for DC60PBS (squares), DC60 (circles), and DPBS (triangles) at *λ* = 633 nm. The lines are guides for the eye. The inset depicts the electric field dependence of the ratio of photoconductivity to dark conductivity, *σ*_p_/*σ*_d_, for the same PR devices at *λ* = 633 nm. The lines are guides for the eye.

From the *σ*_p_ it was possible to determine Φ for the relevant devices and are presented as a function of *E* in Fig. 3. As seen, the Φ are of the same order of magnitude at each *E* for all compositions. The Φ of the DC60 device does consistently exceed that of the DPBS device, suggesting that C_60_ provides superior photosensitization relative to that of QPbS in this composite. With *E* = 100 V/*μ*m, the DC60 shows Φ = 0.30% whereas the DPBS device exhibits Φ = 0.19%. These data indicate, however, that with further optimization, the Φ of QPbS, or some similar material, may exceed that of C_60_. It is also observed that the Φ of the DC60PBS (Φ = 0.44% with *E* = 100 V/*μ*m) device exceeds that of the DC60 device at all *E*. Because the DC60PBS device contains both C_60_ and PbS, and because at the relevant concentrations there is no reason to anticipate any interactions between the C_60_ and the QPbS, to a first approximation, its Φ should be intermediate between that of DC60 and that of DPBS. Therefore, the reason for this observation is not immediately apparent but may indicate an as of yet unknown synergistic effect between the QPbS and the C_60_. The available data do not warrant further speculation.

While the *σ* data provide significant insight into a variety of processes, because the magnitude of the PR space-charge field, |*E*_SC_|, is related to the ratio *σ*_p_/*σ*_d_, these data also show potential for use as a PR material. This relation is provided by the equation


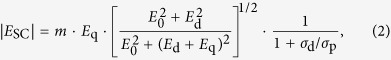


where *E*_0_ is the is the component of *E* which coincides with the grating vector, *m* is the modulation depth, *E*_d_ is the magnitude of the diffusion field, and *E*_q_ is the magnitude of the trap-density-limited space-charge field^24^. The inset of Fig. 3 depicts *σ*_p_/*σ*_d_ as a function of *E*. Interestingly, *σ*_p_/*σ*_d_ reaches a maximum at an intermediate *E*^16^,^17^,^23^. This behavior is highly reminiscent of other Q-dot photosensitized PR composites. However, the device photosensitized with only C_60_, DC60, also shows a similar trend, indicating that this behavior is not unique to composites photosensitized with Q-dots. For the DC60PBS device, the *σ*_p_ exceeds the *σ*_d_ by more than three orders of magnitude, indicating its potential as an efficient PR composite.

**Figure 4:**
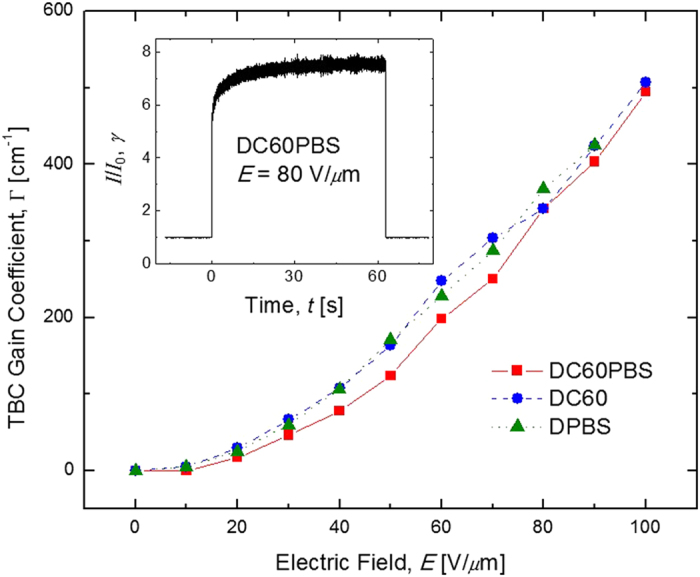
Electric field dependence of the TBC gain coefficient, Γ, for DC60PBS (squares), DC60 (circles), and DPBS (triangles) at *λ* = 633 nm. The lines are guides for the eye. The inset depict the temporal evolution of the experimental quantity *γ* for DC60PBS with *E* = 80 V/*μ*m.

The PR natures of the diffraction gratings were confirmed via the asymmetric exchange of optical energy in conventional TBC experiments. Thi8s exchange of energy is attributed to a spatial shift between the refractive index grating generated in the PR medium and the interference pattern associated with the writing beams^39^. The TBC gain coefficients, Γ, are presented as a function of *E* in Fig. 4. Apparent from the figure is that DC60PBS, DC60, and DPBS exhibit nearly identical Γ across the entire range of *E* investigated. Because the Γ is highly dependent upon the phase shift between the interference pattern and that of the internal space-charge field, which in turn is highly dependent upon the concentration of reasonably deep traps, it can be assumed that the concentration of such deep traps does not vary significantly among the various compositions. This reinforces the hypothesis that narrow band-gap Q-dots have a reduced propensity to act as deep traps in these PR composites relative to their wider-band-gap counterparts. The magnitude of Γ is also noteworthy with DC60 exhibiting Γ = 510 cm^−1^, and DC60PBS showing a slightly diminished Γ = 500 cm^−1^, both at *E* = 100 V/*μ*m. Although the DPBS device experienced dielectric breakdown prior to 100 V/*μ*m, it still showed Γ = 420 cm^−1^ at *E* = 90 V/*μ*m. For practical applications the condition Γ > *α* should be met for a given device. Due to its relatively small *α*_633_, DPBS meets this condition at *E* as low as 10 V/*μ*m. For DC60 and DC60PBS, their Γ exceeds their respective *α*_633 _prior to 20 V/*μ*m. Remarkably, these figures-of-merit are among the best reported for any PR material. The inset in Fig. 4 depicts the optical amplification factor, *γ*, as a function of *t* for the DC60PBS device at *E* = 80 V/*μ*m and provides qualitative insight into the temporal characteristics associated with the TBC in these composites.

**Figure 5:**
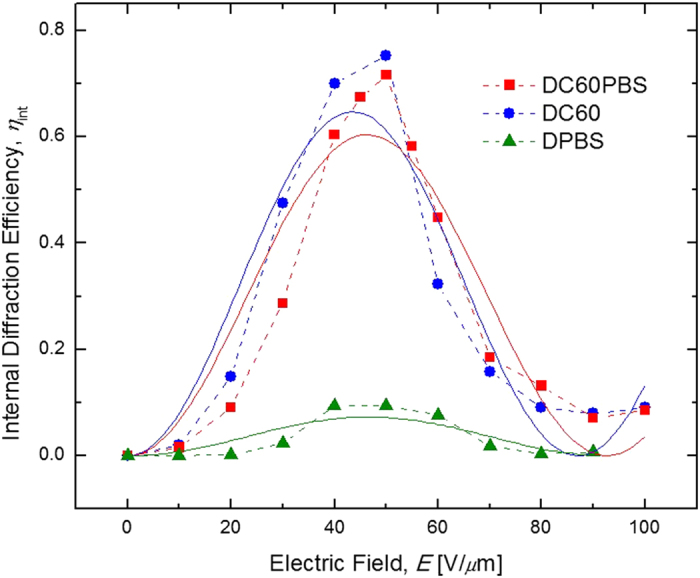
Electric field dependence of the internal diffraction efficiencies, *η*_int_, for DC60PBS (squares), DC60 (circles), and DPBS (triangles) at *λ* = 633 nm. The dashed lines are guides for the eye and the solid lines represent the best fits to a theoretical function (see text).

Having established the PR nature of the observed gratings, the internal diffraction efficiencies, *η*_int_ were evaluated. These data are presented as a function of *E* in Fig. 5. In this figure the solid lines represent the best fit of the data to a simple sine-squared function as predicted by theory^1^. Although the fits are wanting, an over-modulation *E* where *η*_int_ achieves a maximum is clearly observed for all three devices. For DC60PBS and DC60 this occurs at *E*~50 V/*μ*m and for DPBS at *E*~40 V/*μ*m, which are typical values for organic PR composites. Moreover, there exists a subsequent minimum in *η*_int_ for DC60PBS and DC60 at *E*~90 V/*μ*m and for DPBS at *E*~80 V/*μ*m. With *η*_int_ = 75%, it was the DC60 device which exhibited the highest internal efficiency at it over-modulation *E*, however, the addition of the QPbS in the DC60PBS device lowered the efficiency only slightly to 72% at the same *E*. It is central to this study that the diffraction efficiency is maintained in the presence of the QPbS. The performance of the DPBS was far inferior with *η*_int_ = 9.5% at its maximum. However, it is again noted that the composition of this sample was not optimized for performance and is only for comparison purposes, and other studies employing the same QPbS species at higher concentrations in similar composites have shown significantly higher diffraction efficiencies^24^.

**Figure 6:**
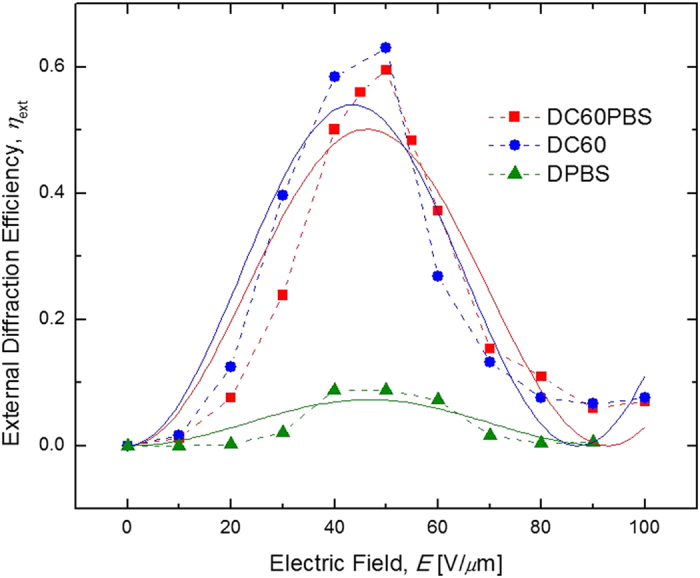
Electric field dependence of the external diffraction efficiencies, *η*_ext_, for DC60PBS (squares), DC60 (circles), and DPBS (triangles) at *λ* = 633 nm. The dashed lines are guides for the eye and the solid lines represent the best fits to a theoretical function (see text).

While the external diffraction efficiencies, *η*_int_ are fundamentally important, it is the *η*_ext_ which are of functional significance and are depicted as a function of *E* in Fig. 6. As in Fig. 5, the solid lines represent the best fit of the data to a simple sine-squared function. Due to their relatively high *η*_int_, in conjunction with their reasonably low *α*_633_, the DC60 and DC60PBS devices also exhibit a favorable *η*_ext_ at their over-modulation *E*, with *η*_ext_ = 63% and *η*_ext_ = 59%, respectively.

**Figure 7:**
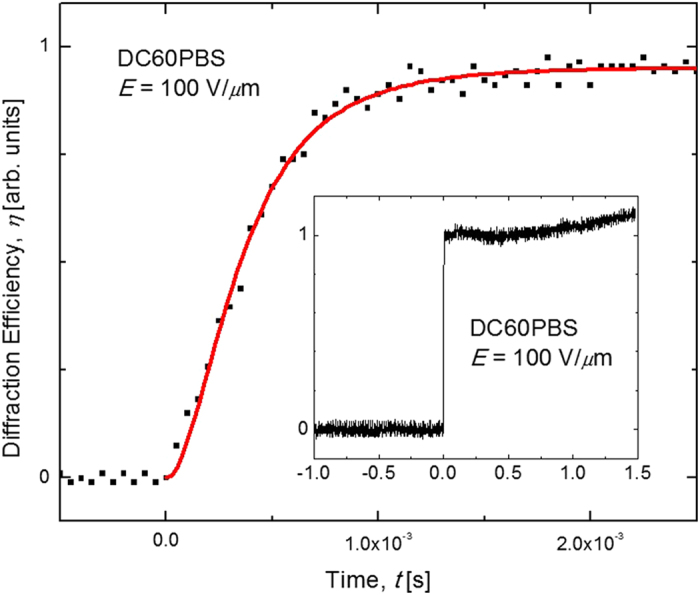
Temporal evolution of the diffracted probe beam, *I*_s_, in the DFWM experiment for the DC60PBS device at *E* = 100 V/*μ*m. The solid line is a fit to a weighted biexponential function (see text). The inset depicts an expanded view of the same data (the fit is eliminated for clarity).

**Figure 8:**
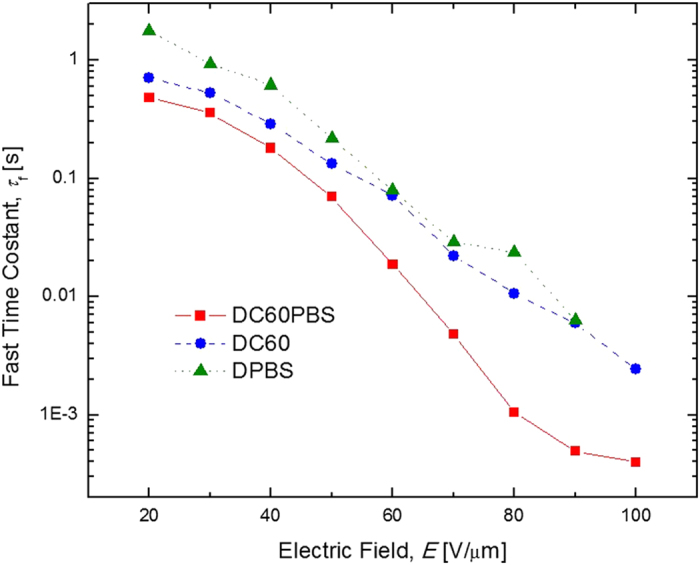
Electric field dependence of the fast time constant, *τ*_f_, for DC60PBS (squares), DC60 (circles), and DPBS (triangles) at *λ* = 633 nm. The lines are guides for the eye.

Time resolved DFWM techniques were used in the quantification of the *τ*. An example of such DFWM data is presented in Fig. 7, where *η* is presented as a function of *t* for the DC60PBS with *E* = 100 V/*μ*m. The solid line in the figure represents the best fit to Eqation 6 and a reasonably good fit to the data is obtained. In this case, the fast time constant, *τ*_f_ = 0.399 ms was derived from the fitting process. This figure-of-merit represents one of the best reported for any PR composite and is nearly an order of magnitude faster than that obtained for the DC60 composite, with *τ*_f_ = 2.42 ms. It is noted that even this *τ*_f_ is among the best for any PR composite under these conditions, which demonstrates the practicality of the DMTPD-based composite. Figure 8 depicts the *τ*_f_ as a function of *E* for the relevant devices. Obvious is that DC60PBS has a significantly faster *τ*_f_ than DC60, and both exhibit superior *τ*_f_ relative to DPBS, for the entire range of *E* investigated. It is speculated that DPBS has a relatively slow *τ*_f_ due to the exceedingly low concentration of photosensitizer present in this composite. As such, it takes longer to produce sufficient number of free charge-carriers to achieve a steady-state space-charge field. These data strongly suggest that the presence of the QPbS has a significant effect on *τ*_f_. These *τ*_f_ indicate the potential for the use of these composites in video-rate applications.

**Figure 9:**
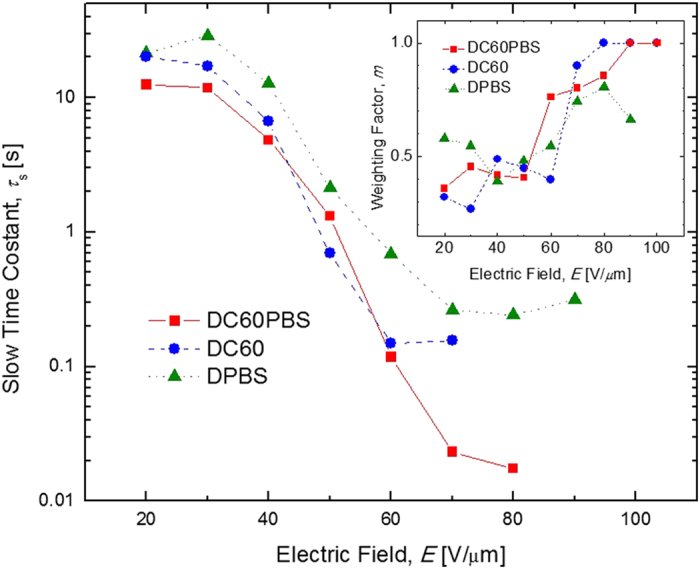
Electric field dependence of the slow time constant, *τ*_s_, for DC60PBS (squares), DC60 (circles), and DPBS (triangles) at *λ* = 633 nm. The lines are guides for the eye. The inset depicts the electric field dependence of the weighting factor, *m*, for the same PR devices. The lines are guides for the eye.

It has been speculated that it is the *τ*_f_ which is associated formation of the space-charge field and it is the slow time constant, *τ*_s_ which is attributed to the reorientation of the NLO chromophore, 7-DCST in this case. The *τ*_s_ are plotted as a function of *E* in Fig. 9. The *τ*_s_ exhibit a similar functional form as the *τ*_f_ however there appears to appear to be more noise in these data. Also evident is that there are no *τ*_s_ reported for DC60PBS for *E* > 80 V/*μ*m or for DC60 when *E* > 70 V/*μ*m. The reason for this is evident from the inset of Fig. 9. As mentioned in the Experimental section, the weighting factor, *m*, was also allowed to float in the fitting process. Apparent from the figure is that the *m* are highly scattered, but display the unmistakable trend of more heavily weighting *τ*_f_ at the expense of *τ*_s_ as *E* is increased for all devices. At relatively low voltages m ≈ 0.5 and the *τ*_s_ and *τ*_f_ contribute almost equally to the grating formation. As higher voltages are attained, the data suggest that it is the formation of the space-charge field which becomes the limiting process. For relatively high voltages *m* tends toward unity and the rise in DFWM signal can be modeled by a single exponential. For DPBS, *m* does increase with *E*, but *m *≠ 1 even at higher *E*. This is again attributed to the relatively slow build up in the density of free charge carriers, and the inability of the NLO chromophore to align quickly in the absence of a reasonably large space-charge field. Though given the scatter in the data, it is best to not draw a strict interpretation concerning this fact.

As outlined in the introduction, QPbS was included in the DC60PBS device with the expectation of enhancing the charge-carrier *μ*, thus improving the *τ* of this PR composite relative to a composite void of QPbS, i.e. DC60. The data, as summarized in Table 2, leave little doubt as to the success of the approach, as *τ*_f_ showed an enhancement of nearly one order of magnitude and *τ*_s_ remained relatively constant. This distinction corresponds to current PR theory in that the presence of the QPbS was included to enhance charge-carrier *μ*, which would directly improve the speed at which the space-charge field is established, and it is this process which is commonly associated with the *τ*_f_. Conversely, there is no reason to expect that the presence of QPbS would influence the rate at which the NLO chromophores, 7-DCST in this case, would reorient within the established space-charge field. Because it this process which is associated with *τ*_s_, it is expected that *τ*_s_ should not be effected by the presence of QPbS and thus remain relatively constant between the DC60PBS and DC60 devices. It is noteworthy that the observed enhancements in *τ*_f_ as well as the various other PR parameters occur at a relatively low concentration of QPbS. While the mechanism responsible for the anticpated improvement in *μ* has been hypothesised in the literature^11^, exactly how such a significant effect can be attributed to such a relativley low concentration of QPbS may not be entirely explained through this proposed mechanism and is the subject of current research. It is further noted that enhancements of similar magnitude have been reported for similarly low Q-dot concentrations^11^,^17^,^18^,^23^.

Although the inclusion of QPbS dramatically improved the *τ*_f_, this observation does not in itself confirm that this enhancement was accomplished through the envisioned mechanism, an enhancement in charge-carrier *μ*, and this conclusion requires some further analysis. Ideally, the *μ* should be measured directly through time-of-flight experiments. An attempt was made in this regard, however, as is typically the case the presence of the highly dipolar 7-DCST resulted in highly diffuse charge-packets precluding such measurements. In an effort to circumvent this issue, samples were fabricated void of 7-DCST however the DMTPD crystalized before such experiments could be completed. Although not possible with the current compositions, previous time-of-flight characterizations conducted with PVK doped with QCdS clearly demonstrated a correlation between the concentration of QCdS and the charge-carrier *μ*^11^. While it is very probable that the observed improvement in *τ*_f_ can be primarily attributed to an enhancement in *μ*, other mechanisms may be considered. Perhaps the most obvious mechanism is one involving the augmentation of charge-carriers with QPbS acting primarily as a photosensitizer. Although QPbS does certainly act as a photosensitizer it is unlikely that such a significant improvement in *τ*_f_ could be attributed to this effect. Although the DPBS device exhibits respectable Φ, the loading content of QPbS, as exemplified by the exceedingly small increase in *α*_633_ in the DPBS device relative to that of the D0 device (2.75 cm^−1^ − 2.46 cm^−1^ = 0.29 cm^−1^), indicates that the QPbS is not likely responsible for any significant increase in the charge-carrier density. Furthermore, if QPbS were significantly contributing to the photo-charge generation process, an accompanying increase in *η* would be expected, but this was not observed. It was also speculated that QPbS may enhance PR performance in ways not related to its ability to act as a photosensitizer. Based on the propensity for Q-dots to act as traps in PR composites, it may be possible that the included QPbS enhances the trapping of holes in the dark fringes. However, because the presence or absence of QPbS in the respective composites had little if any effect on *σ*_d_, and only a favorable effect on *σ*_p_, it seems unlikely that QPbS is acting as a significant trapping species. Interestingly, the fact that the addition of QPbS results in an improvement in *σ*_p_, without affecting *σ*_d_, indicates that enhancements in charge-carrier *μ* may manifest exclusively in illuminated regions and not in dark regions. This performance would be favorable for PR performance because charge-carriers are ideally immobilized within the dark fringes. Lastly, because TBC is highly sensitive to trap density, the similarity in the TBC performances across the various composites further refutes the speculation that QPbS is acting as a significant source of traps.

## Discussion

The goal of this study was to examine the feasibility of using Q-dots to affect charge-carrier *μ* in a PR composite so as to improve the *τ* without detrimentally affecting PR efficiency of the composite. It has been demonstrated that through the inclusion of a narrow bang-gap Q-dot, specifically QPbS, sub-millisecond response times have been achieved for the first time in a PR composite under CW conditions. With a *τ*_f_ = 400 *μ*s, this approach makes the use of PR composites viable materials for video and other high-speed applications. It is noteworthy that the enhancement in *τ*_f_ is not accompanied by a loss in PR efficiency, with *η*_int_ = 72% and Γ = 500 cm^−1^ observed in the same composite, indicating that the included QPbS does not act as a significant of traps. An analysis of the experimental data strongly suggests that the enhancement in *τ*_f_ can be attributed to an improvement in enhanced charge *μ*, which may even be exclusively manifest in the illuminated regions of the PR sample. It is anticipated that this approach can play a significant role in the eventual commercialization of this class of materials.

## Experimental Section

All chemicals were obtained from Aldrich and used as received unless otherwise noted. *QPbS*: QPbS was synthesiszed based on a procedure found in the literature and is described in detail elsewhere^23^,^40^. The QPbS were characterized using visible absorption spectroscopy, thermal gravimetric analysis (TGA), and TEM. The data obtained from these characterizations can also be found elsewhere^23^.

### PR Composite Devices

DMTPD was obtained from Magical Scientific USA and purified by recrystallization in hot CH_2_Cl_2_ prior to use. 7-DCST was synthesized in our lab according to a procedure in the literature^25^. For the composite samples, PVK (secondary standard), DMTPD, 7-DCST and the appropriate quantities of capped QPbS and C_60_ were dissolved in toluene and, after thorough mixing, filtered to remove any undissolved solids. This solution was stored in a vacuum oven at 50 °C for 24 h to remove the solvent. The solid residue was subsequently recovered, placed between two pieces of glass coated with indium tin oxide and heated above its melting temperature on a hot-plate. The sample was then mechanically pressed forming the typical “sandwich” geometry using glass spacers to control the thickness of the device, *d*, at 100 *μ*m.

Although many compositions were considered, the results for four devices are presented here. For these PR devices, the TPD:7-DCST:PVK ratio was held constant at 45:45:10 wt%, respectively. The first PR device contained only these components, constituting a control device and is herein referred to as D0. A second device contained these components in this ratio as well as being photosensitized through inclusion of 0.200 wt % of C_60_. This device is referenced as DC60. A third PR device was fabricated nearly identical to the second with the exception of the addition of 0.223 wt% of QPbS. It is noted that this wt% includes both inorganic PbS as well as the organic capping groups. This device is referred to as DC60PBS. Finally a fourth PR device was fabricated which was identical to the third, with the exception that the C_60_ was not included. This device was fabricated so that the effect of the QPbS over the charge-generation process could be isolated and is referred to as DPBS. To determine the weight percentage of QPbS in each composite, several QPbS:solvent solutions of known absorbance and volume were evaporated to dryness and the mass of the solid residue was measured. It is noted that this residue includes the inorganic PbS as well as the organic passivating layer. The weight percent of QPbS in each device was thus calculated indirectly from the known absorbance and volume of the aliquot used in the fabrication of the respective composite. The compositions and the spectroscopically measured *α*_633_ of all devices are presented in Table 1. The PR devices have not shown any change in their optical properties or degradation in PR performance over the course of 12 months. All UV-Vis absorption spectra were recorded on a Beckman DU 640B spectrophotometer.

### Photorefractive Characterizations

The PR properties of the composite devices were studied via TBC and temporally resolved DFWM techniques using a standard tilted geometry^3^. Holographic gratings were written through the intersection of two coherent beams generated by a helium-neon (HeNe) laser operating at 633 nm with incident angles of *θ*_1_ = 45° and *θ*_2_ = 75° (in air) relative to the sample normal. In the TBC experiments, both writing beams were *p*-polarized with intensities of *I*_1_ ≈ 0.05 mW and *I*_2_ ≈ 8 mW. The external bias was applied such that *I*_1_ would experience gain at the expense of *I*_2_. Asymmetric energy transfer was observed by monitoring the intensities of the writing beams after the PR device with a photodiode. In the DFWM experiment the writing beams were *s*-polarized with intensities of *I*_1_ ≈ 3 mW and *I*_2_ ≈ 9 mW. In addition, a *p*-polarized probe beam propagated in a direction opposite to *I*_1_ with an intensity of *I*_p_ ≈ 2 × 10^−3 ^mW. Through the use of a polarizing beam splitter placed in the path of *I*_2_ in conjunction with a photodiode, the diffracted portion of *I*_p_, also referred to as the signal beam, *I*_s_, could be quantified. In all PR experiments, *I*_1_ and *I*_2_ had beam diameters of ~280 μm while *I*_p_ possessed a beam diameter of ~ 120 μm. Beam diameters were measured by using a fractional irradiance of 1/e^2^.

The Γ, was determined in terms of the experimentally measured quantities *γ* and *β*, as


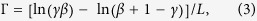


where *L* is the path length of the beam experiencing gain inside the sample, *β* is the ratio of the writing beam intensities before the sample, and *γ* is the ratio of the intensity of the beam experiencing gain with and without the pump beam. The *η*_int_ were quantified according to the equation


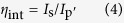


where *I*_p′_ is the intensity of the probe beam after the device with no bias applied. Similarly, the *η*_ext_, accounting for reflections, were determined according to the equation


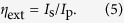


Time resolved DFWM techniques were used in the quantification of the *τ*. For this experiment, *E* was applied to the device while blocking one of the writing beams. The device was permitted to settle for approximately 30 s and the blocked writing beam was unblocked. The diffracted portion of the probe beam was then recorded as a function of time, *t*. The determination of the *τ* was accomplished by fitting the data obtained in DFWM experiments to an equation of the form


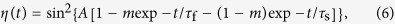


where *A* is a fitting constant, *m* is a weighting ratio, *τ*_f_ is the fast time constant and *τ*_s_ is the slow time constant. For the fitting, all four variables were allowed to float.

Conductivity characterizations were made using a dc-photocurrent technique with a Keithley electrometer used to measure the current passing through the sample as a function of *E*. The beam intensity for all *σ*_p_ characterizations was ~10 mW with a beam diameter of 0.98 mm. To measure the *σ*_p_, the sample was initially illuminated for several minutes followed by the application of the *E* and the current was allowed to achieve a steady state (typically ~5 min). To measure the dark conductivity, *σ*_d_, an *E* was applied and the current was allowed to reach a steady state. In calculating the *σ*_p_, the dark current density was subtracted from the photocurrent density. For calculations involving the photocurrent density, the diameter of the laser was employed, and for those related to the dark current density, the area of electrode overlap was used. These data were calculated using the equation


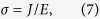


where *J* is the experimentally determined current density.

From the *σ*_p_ data it is possible to determine the internal photocurrent efficiency, Φ, of the photosensitizer using the equation


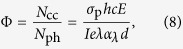


where *N*_cc_ is the number of charge-carriers generated per unit volume, *N*_ph_ is the number of photons absorbed per unit volume, *h* is Plank’s constant, *c* is the speed of light, and *e* is the fundamental unit charge.

## Additional Information

**How to cite this article**: Moon, J.-S. *et al*. Sub-Millisecond Response Time in a Photorefractive Composite Operating under CW Conditions. *Sci. Rep.* **6**, 30810; doi: 10.1038/srep30810 (2016).

## Figures and Tables

**Table 1:** Compositions and *α*_633_ of the photorefractive devices used in this study.

Device	C_60_ (wt%)	QPbS (wt%)	DMTPD (wt%)	7-DCST (wt%)	PVK (wt%)	*α*_633_ (cm^−1^)
DC60PBS	0.199	0.223	44.8	44.8	9.96	8.04
DC60	0.200	NA	44.9	44.9	9.98	7.81
DPBS	NA	0.223	44.9	44.9	9.98	2.75
D0	NA	NA	45.0	45.0	10.0	2.46

**Table 2:** Summary of the PR figures-of-merit attributed to the various composites relevant to this study.

Device	*τ*_f_ (ms)	*τ*_s_ (ms)	*η*_int_ (%)	*η*_ext_ (%)	Γ (cm^−1^)	Γ-*α*_633_ (cm^−1^)
DC60PBS	0.40 ± 0.01^a^	17.5 ± 0.7^c^	72^e^	59^e^	500^a^	492^a^
DC60	2.4 ± 0.2^a^	156 ± 3^d^	75^e^	63^e^	510^a^	502^a^
DPBS	6.3 ± 0.1^b^	310 ± 50^b^	9.5^f^	8.9^f^	420^b^	417^b^

^a^*E* = 100 V/*μ*m.

^b^*E* = 90 V/*μ*m.

^c^*E* = 80 V/*μ*m.

^d^*E* = 70 V/*μ*m.

^e^*E* = 50 V/*μ*m.

^f^*E* = 40 V/*μ*m.
